# The Scent of Antifungal Propolis

**DOI:** 10.3390/s21072334

**Published:** 2021-03-27

**Authors:** Zsigmond Papp, Sarra Bouchelaghem, András Szekeres, Réka Meszéna, Zoltán Gyöngyi, Gábor Papp

**Affiliations:** 1 Department of Public Health Medicine, Medical School, University of Pécs, Szigeti út, 12, 7624 Pécs, Hungary; toxingmoon@gmail.com; 2 Department of General and Environmental Microbiology, Institute of Biology, University of Pécs, Ifjúság u, 6, 7624 Pécs, Hungary; bouchelaghem24sarra@gmail.com (S.B.); meszenareka@gmail.com (R.M.); pappgabor.pappgabor@gmail.com (G.P.); 3 Department of Microbiology, Faculty of Science and Informatics, University of Szeged, Közép fasor 52, 6726 Szeged, Hungary; andras.j.szekeres@gmail.com

**Keywords:** antifungal, propolis, gas chromatography, classification, electronic nose, *Candida albicans*

## Abstract

Propolis contains many effective antifungal compounds that have not yet been identified and evaluated. In addition, distinguishing samples of propolis with high antifungal activity from less active ones would be beneficial for effective therapy. Propolis samples were collected from four different geographical regions in Hungary and used to prepare ethanol extracts for analysis. First, an antifungal susceptibility test was performed on *Candida albicans*. Then, gas chromatography-mass spectrometry (GC-MS) and an opto-electronic nose were applied for the classification of propolis samples. In three propolis samples, the IC_50_ was measured between 72 and 134 µg/mL, but it was not calculable in the fourth sample. GC-MS analysis of the four propolis samples identified several compounds belonging to the various chemical classes. In the antifungal samples, the relative concentration of 11,14-eicosadienoic acid was the highest. Based on the opto-electronic electronic nose measurements, 98.4% of the original grouped antifungal/non-antifungal cases were classified correctly. We identified several molecules from propolis with potential antifungal properties. In addition, this is the first report to demonstrate the usefulness of a portable opto-electronic nose to identify propolis samples with high antifungal activity. These results may contribute to the rapid and efficient selection of new fungicide-candidate molecules and effective propolis samples for treatment.

## 1. Introduction

Propolis is a generic name for an adhesive material gathered by honeybees to maintain and protect their hive [1]. Evidence for the use of propolis has existed since 300 BC, and it has been used thereafter for various purposes: in dermatological products to treat various skin conditions (e.g., psoriasis and herpes simplex), to treat rheumatism and sprains, and in dental medicine as an anaesthetic agent and possible treatment for gingivitis, cheilitis, and stomatitis [2]. Propolis has also been demonstrated to have hepatoprotective, antitumor, anti-inflammatory, and antimicrobial properties [3].

Several studies have aimed to collect data concerning the antifungal properties of propolis, and the results were positive; however, differences in the antifungal properties were observed in different propolis samples because of their varying chemical composition, geographical origin, season of collection, flora, and method of extraction [4,5]. Propolis has antifungal activity alone [6,7] but also functions synergistically when used simultaneously with other antifungal agents against *Trichophyton*, *Mycrosporum,* and *Candida albicans* [8]. Further, a study using propolis extract showed antifungal activity towards fungi in both the planktonic form and in biofilms [9]. Using propolis as a topical antifungal agent or complementary substance in antifungal medicines has been considered [2,9] because the cytotoxicity of propolis can be several times lower than that of synthetic antifungal substances [10].

Flavonoids, phenolics, and aromatics are the main pharmacologically active components in propolis, and flavonoids account for most of its biological activities [5]. Lignans, terpenes, fatty acids, sugars, hydrocarbons, cinnamic acids, and p-coumaric acids are also found in propolis samples. Volatile compounds can be used to characterise the propolis samples by their region of origin [1,5,11,12]. To identify the differences in the chemical contents of propolis samples originating from various regions, gas chromatography-mass spectrometry (GC-MS) analysis of the trimethylsilyl (TMS)-derivatised components is a frequently used technique that can measure the relative amount of each chemical present in a given sample [13,14,15].

Electronic noses with sensor arrays can detect volatile molecules; however, an activity pattern of single sensors can be used to identify specific scents. Among the latest developments, peptides are used as sensors, mimicking human and animal olfactory systems [16]. To classify different scents, appropriate data processing should be chosen. The unsupervised machine learning algorithm principal component analysis (PCA) is widely applied for electronic noses, but the supervised linear discriminant analysis (LDA) provides better classification [17].

The full spectrum of compounds in propolis samples is presumably incomplete. New substances in propolis samples must be identified to reveal the connection between antifungal activity and chemical composition, helping to decide whether a propolis sample can be used in antifungal therapy. If the chemical composition and antifungal activity are known, the volatile compounds can help establish a link between the scent of the sample and its antifungal properties. Using scent to collect information about the antifungal properties has potential as a fast analysis method for propolis. To develop this method, we collected poplar-type propolis samples with lower and higher antifungal activity, analysed the propolis extracts via GC-MS, and used a peptide-based opto-electronic nose to classify the samples.

## 2. Materials and Methods

### 2.1. Origin of Raw Propolis and Preparation of Ethanol Extracts

Raw poplar-type propolis samples were collected in 2015 from four regions of Hungary: Csikóstőttős (CS), Héhalom (HE), Somogybabod (SO), and Szolnok (SZ). The samples were ground, and 100 g of propolis was extracted in 450 mL of 80% ethanol in a water bath at 70 °C for 30 min. The ethanol extracts were sterilised through a 0.22 µm pore size filter (Millipore, Burlington, MA, USA) to obtain a 222.2 mg/mL stock concentration. The ethanol extracts of propolis (EEPs) were stored at 4 °C in the dark [18].

### 2.2. Antifungal Susceptibility Testing of C. albicans

The susceptibility of *C. albicans* ATCC 44829 to four EEPs was determined according to the CLSI M27-A2 [19] standard broth microdilution method keeping 80% (*v*/*v*) ethanol concentration of stock solution. The control was 80% (*v*/*v*) ethanol. The stock solution was 1% of the culture medium resulting in a 0.8% (*v*/*v*) final concentration of ethanol in both treated and control solutions. The cell culture was maintained on YEPD-agar plates (0.5% (*w*/*v*) yeast extract, 2% (*w*/*v*) glucose, 1% (*w*/*v*) bacteriological peptone, 2% (*w*/*v*) agar, supplemented with 25 mg/L of adenine; pH 5.6). Briefly, two-fold serial dilutions of EEP (6.25–400 µg/mL) were mixed in a 1:1 ratio with fungal suspensions in RPMI-1640 medium buffered with 0.165 mol/L of MOPS (pH 7.0). The cell number in the mixture was adjusted to a final concentration of 2.5 × 10^3^ cells/mL in 96-well cell culture plates (REF3595; Costar^®^, Kennebunk, ME, USA) and incubated at 35 °C for 48 h. The solvent concentration was kept constant (1%) in any given well. The absorbances of the suspensions proportional to growth were measured in the wells at 595 nm using a Multiskan EX plate reader (Thermo Fisher Scientific, Waltham, MA, USA). All of the experiments were repeated three times. The minimum inhibitory concentration (MIC) values were determined as the lowest concentration at which 80% growth inhibition occurred [20].

### 2.3. GC-MS Analysis of the Propolis Extracts

The TMS ether derivatives of ethanolic extracts of propolis were subjected to GC-MS analysis. Briefly, about 2.2 mg of freeze-dried EEP was mixed with 50 μL of dry pyridine (Merck, Budapest, Hungary) and 75 μL of MSTFA (Merck, Hungary) and heated at 80 °C for 20 min. GC-MS analysis of the derivatised samples was performed using a QP-2020 GC-MS system (Shimadzu, Duisburg, Germany) equipped with a 30 m long, 0.25 mm i.d. and 1 μm film thickness DB5-MS (Agilent, Santa Clara, CA, USA) capillary column. The temperature was programmed from 100 to 320 °C at a rate of 5 °C/min. Helium was used as a carrier gas at a flow rate of 40 cm/s. The split ratio was 1:20, the injector temperature was 280 °C, and the interface temperature was 320 °C. For MS, the EI (electron ionization) ion source temperature was 230 °C, the ionisation voltage was 70 eV, and the solvent cut time was 4.0 min. The data were recorded in the scan mode with a 0.3 s event time from 4.5 to 60 min in the 45–600 *m*/*z* range. A hexane solution of C7–C33 n-alkanes (Restek, Bellefonte, PA, USA) was also separated under the above conditions for the retention index calculations. The identification of individual compounds by GC-MS was performed by searching for their mass spectra separately against the NIST 17 and Smart Metabolites libraries using the internal library search algorithm for the Shimadzu GC-MS Solutions V.4.45 software (Shimadzu, Duisburg, Germany).

### 2.4. Classification of Propolis Extracts Using an Electronic Nose

The propolis samples were separated by region. Working stock solutions were made from each propolis sample: 15 µL of EEP, 34 µL of 96% ethanol, and 4150 µL of distilled water were mixed and homogenised to produce stock solutions of 800 µg/mL. From each stock solution, 8 × 500 µL was measured in vials. For the control sample, 83 µL of 96% ethanol and 7920 µL of distilled water were mixed for the stock solution. From this stock solution, 15 × 500 µL was used to measure the headspace in 20-mL vials.

First, the ethanol control samples were measured using the NeOse Pro electronic nose system (Aryballe Technologies, Grenoble, France); the opto-electronic sensor array uses 63 non-specific peptides printed on a gold layer [21]. Dynamic measurement was used as follows: the pump flow rate was set to 40 mL/min; the number of frames per second was 20; the environmental temperature was 29 °C; the core temperature was 44 °C; the humidity level was 25%. After measuring the ethanol control samples, the propolis samples were measured using the same settings.

### 2.5. Statistics

To determine significant differences in antifungal activity among the propolis samples, we compared the survival percentages with that of the non-antifungal (SO) sample, applying Student’s *t*-test (* *p* < 0.05, ** *p* < 0.01, and *** *p* < 0.001). The lowest significance level from the three samples compared with the SO sample was labelled in each concentration. To compare the propolis samples in GC-MS, we calculated the ratio of the area of identified chemicals in the non-antifungal (SO) sample and the mean of the areas of the corresponding chemical in all of the antifungal (SZ, HE, and CS) samples. For the signals collected from 63 individual sensors from each measurement using the opto-electronic nose, LDA was computed using IBM SPSS Statistics for Windows, Version 26 (IBM, Armonk, NY, USA), in which Fisher’s coefficient and the Mahalanobis distance were used for stepwise analysis.

## 3. Results

### 3.1. Antifungal Susceptibility

**Figure 1:**
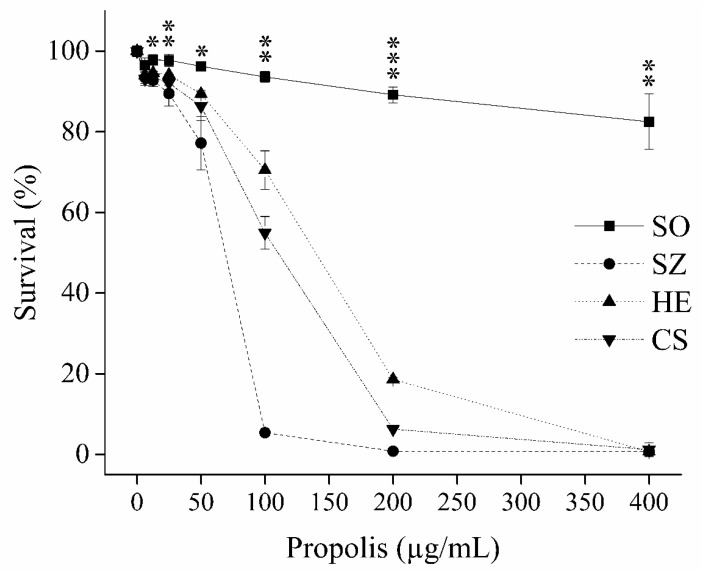
Inhibitory curve. Survival of *C. albicans* ATCC 44,829 after 48 h of incubation at 35 °C (y-axis), using 0, 6.25, 12.5, 25, 50, 100, 200, and 400 µg/mL of different ethanol extract of propolis (EEP) samples (x-axis) from diverse geographical areas in Hungary: Somogybabod (SO), Szolnok (SZ), Héhalom (HE), and Csikóstőttős (CS). The treated and control samples contained a final ethanol concentration of 0.8% (*v*/*v*), and the Student’s *t*-test was applied to calculate the level of significance between low antifungal activity (SO) and high antifungal activity samples (SZ, HE, and CS). The figure illustrates the lowest level of significance among the low antifungal (SO) and high-antifungal (SZ, HE, and CS) samples. * *p* < 0.05, ** *p* < 0.01, and *** *p* < 0.001.

The cytotoxicities of EEPs were characterised by determining the antifungal susceptibility of *C. albicans* cells after 48 h of using the microdilution method. All the extracts showed concentration-dependent susceptibility (Figure 1). The SZ, HE, and CS samples had potent antifungal activity and showed an MIC_80_ and IC_50_ in the range of 100–200 μg/mL and 72–134 µg/mL, respectively (Table 1). Although the SO sample was significantly weaker than that of the other EEP samples, its effect on reducing the growth of the cells did not exceed 18% at 400 μg/mL.

### 3.2. GC-MS Analysis

GC analysis identified 148 components in the EEPs, among which 134 were detectable in the HE sample, 115 in the CS sample, 127 in the SZ sample, and 94 in the SO sample. Using the chemical structures, the components could be classified as alcohols, aliphatic and aromatic aldehydes, alkanes, amino acids, aliphatic and aromatic carboxylic acids, essential oils, esters, fatty acids, fatty alcohols, flavonoids, ketones, polyphenols, sugars (monosaccharides, disaccharides), sugar acids, sugar alcohols, phenols, phenolic acids, terpenes, terpene alcohols, vitamin B6, and other structures such as heptalene and urea.

Among the antifungal samples, the five most abundant components were chrysin (25.65%; polyphenolic flavone/flavonoid), genistein (21.69%; isoflavone), ethyl gallate (6.62%; carboxylic acid), caffeic acid (5.69%; cinnamic acid), and caffeic acid ethyl ester (4.22%; hydroxycinnamic acid/polyphenol); in the less active antifungal sample, the five most abundant components were chrysin (19.01%; flavonoid), D-fructofuranose-pentakis(trimethylsilyl) ether (isomer; psicose) (16.46%; monosaccharide), genistein (13.83%; flavonoid), sucrose (10.54%; disaccharide), and alpha-D-glucopyranose (6.44%; monosaccharide).

The ratios of the substances in the active and less active antifungal samples were determined. Table 2 lists the chemicals constituting the antifungal samples (HE, CS, SZ) with a more than five times higher concentration than the less active antifungal sample (SO).

### 3.3. Electronic Nose

**Figure 2:**
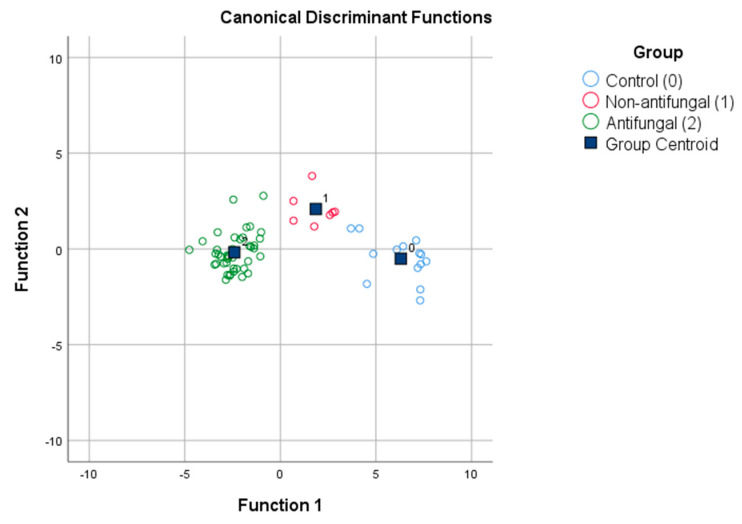
LDA. Linear discriminant analysis (LDA) classifies EEP samples by their low or high antifungal activity and control ethanol solvent. The original data were obtained using the NeOse Pro opto-electronic nose with 63 different sequences of peptides on a sensor array.

The results of the measurements using the electronic nose were analysed by IBM SPSS Statistics. Three groups were created, one each for the ethanol control, antifungal samples, and samples with less active antifungal properties. Discriminant analysis was used to determine whether the different groups separated from each other. Figure 2 shows marked separation of the different samples: the control samples, samples with antifungal properties, and samples with less active antifungal properties are clearly detached from each other, making distinct group centroids. The stepwise method resulted in correct classifications in 98.4% of original grouped cases but, using cross-validation, 95.2% of grouped cases were classified correctly (Table 3).

## 4. Discussion

During the last decade, propolis is among the natural substances that has attracted the attention of researchers because of its various therapeutic properties. Several studies have described the biological activity of propolis as an antifungal agent. However, the floral sources of propolis samples can affect their efficacy [22].

This study aimed to characterise and compare four ethanol extracts of poplar-type propolis collected from different regions in Hungary without overlapping the bee territories. Surprisingly, one of the samples had significantly lower antifungal activity, whereas the other samples had high antifungal activity against *C. albicans*.

GC-MS analysis revealed a substantial difference between samples with or without antifungal properties. Among the main components, chrysin was recognised as an active growth inhibitor of *Candida* [23]. Cinnamic acid derivatives were more abundant in samples with higher antifungal activity. This compound has also been reported to inhibit fungal growth [24]. Genistein acts primarily as an antioxidant phytoestrogen without observable fungicide properties [25]. Similarly, ethyl gallate shows little antifungal effect, but its synergistic activity is detectable [26].

In contrast to the samples with high antifungal activity, the main components of samples without a significant antifungal effect were dominantly genistein and saccharoses, which have no significant fungicide effects reported in the literature.

The other strategy was to calculate the rate of components in antifungal and non-antifungal samples. A literature search revealed that some of the identified molecules had no reported antifungal activity. In the other category, identifying a direct association was challenging because no study is available using purified substances, only natural mixtures. However, farnesol [27], vanillin [28], 4-coumaric acid [29], and methyl ferulate [30] are among molecules reported to be good inhibitors of biofilm formation in microorganisms, including *C. albicans*.

Three categories of identified molecules in the samples are characteristically found in propolis samples with high antifungal activity. The first category contains components with known antifungal activity. In the second category, molecules act synergistically; in the third group, molecules have no known antifungal activity. The latter group of chemicals can be divided further into subgroups: (i) with no real antifungal effect and (ii) with a real but unproven antifungal effect.

In addition to suggesting new target molecules for antifungal studies, our main goal was to identify propolis samples with high antifungal activity to improve antifungal propolis therapy. Electronic noses are sparsely used tools in the classification of biological samples because they are a relatively new technology in the field of measurement. Cheng et al. [31] reported the successful classification of propolis samples by geographical region using the Alpha M.O.S. electronic nose (Heracles, France), but they did not analyse the antifungal activity of the samples. For classification, we applied an opto-electronic nose, NeOse Pro, using the LDA machine learning algorithm. The tested opto-electronic nose using a peptide sensor array and a chosen data processing algorithm classified propolis samples with more than 95% accuracy according to their antifungal capacity. This is the first study to classify a potentially therapeutic substance, revealing that opto-electronic noses can be used in the medical field.

## 5. Conclusions

Various antifungal properties of poplar-type propolis samples originate from different geographical regions. Using GC-MS, our study demonstrated that the antifungal activity of propolis samples is associated with the abundance of antifungal components. Using an opto-electronic nose, we classified first propolis samples according to their high or low antifungal activity. The classification accuracy was over 95%. These results may contribute to the identification of new antifungal molecules and rapid selection of effective antifungal propolis samples for treatment.

## Figures and Tables

**Table 1:** Inhibitory data. The minimum inhibitory concentration (MIC) values were determined as the lowest concentration at 80% growth inhibition. The half-maximal inhibitory concentration (IC_50_) values were also determined using four different ethanol extracts of propolis on *C. albicans*. The values are expressed in µg/mL. The geographical areas of propolis origin were Somogybabod (SO), Szolnok (SZ), Héhalom (HE), and Csikóstőttős (CS).

EEP (µg/mL)	SO	SZ	HE	CS
IC_50_	No	72	134	108
MIC_80_	No	100	200	200

**Table 2:** Relative dominance of chemicals in antifungal samples. The ratios of chemical concentrations (high-antifungal-activity/low-antifungal-activity sample) showing more than 5 times higher concentration in antifungal samples. The original data were obtained from GC-MS measurement and calculated from the area values.

Component	>5-Times Higher Concentration in Antifungal Samples [Times]
11,14-Eicosadienoic acid	16.84
Ferulic acid	14.87
Benzene propanoic acid	13.13
Farnesol	12.99
Cinnamic acid	12.97
Urea	12.16
Benzoic acid	11.66
17-Octadecynoic acid	10.96
alpha/beta-Eudesmol	10.90
Vanillin	9.97
Ricinoleic acid	8.88
4-Methoxycinnamic acid	8.64
cis/trans p-Coumaric acid	8.60
Benzyl alcohol	8.21
cis/trans p-Coumaric acid	8.10
Hexadecyl-p-coumarate	7.30
1,3,5-Benzetriol	7.03
Coniferyl aldehyde	7.02
Isoferulic acid	6.89
Pyridoxine	6.79
Methyl ferulate	6.55
Propanoic acid	6.19
alpha/beta-Eudesmol	5.67
Methyl 2-amino-3-hydroxybenzoate	5.46
Caffeic acid	5.07
Caffeic acid, ethyl ester	5.06

**Table 3:** Classification results. Original and cross-validated classification of propolis samples with very low antifungal (non-antifungal) or high antifungal (antifungal) capacity are displayed with the vehicle control (control). The samples were classified by linear discriminant analysis (LDA).

Classification Results ^a, c^						
		Predicted Group Membership				
		Group	Control	Non-Antifungal	Antifungal	Total
Original	Count	Control	13	1	0	14
		Non-antifungal	0	7	0	7
		Antifungal	0	0	42	42
	%	Control	92.9	7.1	0	100.0
		Non-antifungal	0	100.0	0	100.0
		Antifungal	0	0	100.0	100.0
Cross-validated ^b^	Count	Control	12	2	0	14
		Non-antifungal	0	7	0	7
		Antifungal	0	1	41	42
	%	Control	85.7	14.3	0	100.0
		Non-antifungal	0	100.0	0	100.0
		Antifungal	0	2.4	97.6	100.0

^a^. 98.4% of original grouped cases correctly classified. ^b^. Cross-validation was undertaken only for those cases in the analysis. In cross-validation, each case is classified by the functions derived from all cases other than that case. ^c^. 95.2% of cross-validated grouped cases correctly classified.

## Data Availability

The data presented in this study are available on request from the corresponding author.
